# Comparative Analysis of Calcium-Dependent Protein Kinase in Cucurbitaceae and Expression Studies in Watermelon

**DOI:** 10.3390/ijms20102527

**Published:** 2019-05-23

**Authors:** Chunhua Wei, Ruimin Zhang, Xiaozhen Yang, Chunyu Zhu, Hao Li, Yong Zhang, Jianxiang Ma, Jianqiang Yang, Xian Zhang

**Affiliations:** State Key Laboratory of Crop Stress Biology in Arid Areas, College of Horticulture, Northwest A&F University, Yangling 712100, China; xjwend020405@nwafu.edu.cn (C.W.); zrm0923@163.com (R.Z.); yxzh5186@126.com (X.Y.); zhucy@nwafu.edu.cn (C.Z.); yuanyilihao123@163.com (H.L.); zhangyong123@nwafu.edu.cn (Y.Z.); majianxiang@126.com (J.M.); yangjq1208@126.com (J.Y.)

**Keywords:** calcium-dependent protein kinases, CDPK-related kinases, evolutionary analysis, expression pattern, abiotic stress, cucurbitaceae

## Abstract

Both the calcium-dependent protein kinases (CDPKs) and CDPK-related kinases (CRKs) play numerous roles in plant growth, development, and stress response. Despite genome-wide identification of both families in *Cucumis*, comparative evolutionary and functional analysis of both *CDPKs* and *CRKs* in Cucurbitaceae remain unclear. In this study, we identified 128 *CDPK* and 56 *CRK* genes in total in six Cucurbitaceae species (*C. lanatus*, *C. sativus*, *C. moschata*, *C. maxima*, *C. pepo*, and *L. siceraria*). Dot plot analysis indicated that self-duplication of conserved domains contributed to the structural variations of two *CDPKs* (*CpCDPK19* and *CpCDPK27*) in *C. pepo*. Using watermelon genome as reference, an integrated map containing 25 loci (16 *CDPK* and nine *CRK* loci) was obtained, 16 of which (12 *CDPK* and four *CRK*) were shared by all seven Cucurbitaceae species. Combined with exon-intron organizations, topological analyses indicated an ancient origination of groups CDPK IV and CRK. Moreover, the evolutionary scenario of seven modern Cucurbitaceae species could also be reflected on the phylogenetic trees. Expression patterns of *ClCDPKs* and *ClCRKs* were studied under different abiotic stresses. Some valuable genes were uncovered for future gene function exploration. For instance, both *ClCDPK6* and its ortholog *CsCDPK14* in cucumber could be induced by salinity, while *ClCDPK6* and *ClCDPK16*, as well as their orthologs in *Cucumis*, maintained high expression levels in male flowers. Collectively, these results provide insights into the evolutionary history of two gene families in Cucurbitaceae, and indicate a subset of candidate genes for functional characterizations in the future.

## 1. Introduction

As a ubiquitous second messenger, Calcium (Ca^2+^) plays an important role in sophisticated signal transduction pathways to survive frequently occurring environmental stresses during plant growth and development [1,2]. Transient changes of the Ca^2+^ concentration in the cytoplasm can be sensed by four types of calcium sensors: calmodulins (CaM), calmodulin-like proteins (CaML), calcineurin B-like proteins (CBL), and the calcium-dependent protein kinase (CDPK) [3,4]. Among these sensors, CDPKs not only sense but also directly translate Ca^2+^ signals into a downstream phosphorylation pathway, thus functioning both as Ca^2+^ sensors and effectors [2,5,6].

The CDPKs (also referred to as CPKs) are ser/thr protein kinases, which consist of four typical domains: a variable N-terminal domain (containing the myristoylation and palmitoylation sites), a catalytic ser/thr protein kinase domain, an auto-inhibitory domain (acting as a pseudosubstrate combined with the kinase domain to inhibit activity), and a C-terminal regulatory calmodulin-like domain that contains one to four EF-hand motifs for Ca^2+^ binding [7,8,9]. CDPK-related kinases (CRKs) have similar domain structures than CDPKs (such as the ser/thr kinase domain); however, they do not have EF-hand domains [10,11]. To date, genome-wide identification of *CDPKs* has been widely performed in a large number of plants, e.g., 34 *CDPKs* have been identified in *Arabidopsis* [7], 31 in rice [12], 29 in tomato [10], 18 in melon [11], and 19 in cucumber [9]. Similarly, *CRKs* have also been identified in genomes, e.g., eight *CRKs* in *Arabidopsis* [13], five in rice [14], six in tomato [10], five in pepper [15], and seven in melon [11].

Accumulating evidence indicates that *CDPK* and *CRK* genes are not only involved in plant growth and development, but also in the plant response to abiotic and hormone stresses. For example, AtCPK32 in *Arabidopsis* has been reported to interact with the calcium channel protein CNGC18, thus controlling the polar growth of pollen tubes [16]. In addition, *AtCPK4* and its homolog *AtCPK11* have been reported to function in seed germination and growth, stomatal movement, and response to salt stress [17]. The gene *AtCPK23* acts as a negative regulator and plays important roles in response to drought and salt stresses via controlling K^+^ channels; its overexpression can increase stomatal apertures [18]. In grape plants, a large number of *CDPKs* have been reported to be induced in response to various abiotic and biotic stresses, as well as to hormone treatments [19]. A previous study showed that the *CDPK* genes in cucumber are extensively regulated by various stimuli, including salt, cold, heat, waterlogging, and abscisic acid stresses, possibly following different mechanisms [9]. Furthermore, *CmCDPKs* and *CmCRKs* in melon were also shown to be differentially expressed in response to exogenous stresses, such as biotic stress (*Podosphaera xanthii* inoculation), abiotic stresses (salt and cold), and hormone (abscisic acid) treatment [11]. In general, *CDPK* genes are ubiquitously expressed in most of the different plant organs. However, several genes show organ- or tissue-specific expression patterns. For instance, *VvCDPK5* in grape plants can only be detected in pollen [20]. Similarly, *AtCPK17* and *AtCPK34* are both preferentially expressed in mature pollen, regulating the growth of pollen tubes [21].

The Cucurbitaceae family contains several economically important species with already published genomes, including watermelon (*Citrullus lanatus*), melon (*Cucumis melon*), cucumber (*Cucumis sativus*), and bottle gourd (*Lagenaria siceraria*), which belong to the Benincaseae tribe and three *Cucurbita* species (*Cucurbita maxima*, *Cucurbita moschata*, and *Cucurbita pepo*) of the Cucurbiteae tribe [22,23,24,25,26,27]. Although genome-wide identifications of *CDPKs* and *CRKs* have been performed in the genus *Cucumis* [9,11], comparative evolutionary analysis of both gene families in Cucurbitaceae is lacking. In this study, we identified a total of 128 *CDPK* and 56 *CRK* genes in six Cucurbitaceae species (*C. lanatus*, *C. sativus*, *C. moschata*, *C. maxima*, *C. pepo*, and *L. siceraria*). After mapping these identified genes onto chromosomes of watermelon, we obtained an integrated map including 25 loci (16 *CDPK* and nine *CRK* loci). Evolutionary analyses revealed that four CDPK groups and one CRK group in phylogentic trees could be further divided into loci, consistent with the integrated map. In addition, expression patterns of *ClCDPKs* and *ClCRKs* under different abiotic stresses were also analyzed. Our results provide insights into the evolutionary history of both gene families in Cucurbitaceae, and indicate a subset of candidate genes for future functional analysis.

## 2. Results

### 2.1. Genome-Wide Identification of CDPK and CRK Genes

Our previous study verified 18 *CmCDPK* and seven *CmCRK* genes in melon [11], while only 19 *CDPK* homologs were identified genome-wide in cucumber [9]. Hence, to comparatively analyze the *CRK* gene family in Cucurbitaceae, identification of *CRK* genes in cucumber genome was also performed in this study. As a result, a total of 128 *CDPK* (typically containing both STKs_CAMK protein kinase and EF-hand domains) and 56 *CRK* (solely harboring STKs_CAMK protein kinase domain) genes were identified in six Cucurbitaceae species (*C. lanatus*, *C. sativus*, *C. moschata*, *C. maxima*, *C. pepo*, and *L. siceraria*). All the identified genes were designated based on their chromosomal locations (Table S2). It is worth noting that two *CDPKs*, referred to as *CsCDPK18* (*Csa018149*) and *CsCDPK15* (*Csa008536*) in the previous study [9], were renamed to *CsCRK6* and *CsCRK7* due to their lack of EF-hand domains. Compared to the similar copy numbers of *CDPK* and *CRK* genes in four species (*C. lanatus*, *C. melon*, *C. sativus*, and *L. siceraria*) of the Benincaseae tribe (Table 1), many more homologs were identified in three Cucurbiteae tribe genomes (*C. moschata*, *C. maxima*, and *C. pepo*). This may have been caused by the whole-genome duplication (WGD) that only occurred in the progenitor of the *Cucurbita* genus [25,26].

Physico-chemical properties of CDPKs and CRKs, including predicted amino acids, molecular weight (MW), and isoelectric point (pI), showed similar ranges in watermelon, melon, and cucumber; however, these characteristics exhibited broader intervals in the remaining four species (Table 1). In this study, six CDPKs were predicted to have long (>1000) amino acid sequences, possibly due to their long CT domains [28]. Compared to CRKs with no EF-hand domain, the majority of CDPKs were predicted to contain four EF-hands, with few exceptions harboring two or three EF-hand motifs (Table S2). Strikingly, two CDPKs (CpCDPK19 and CpCDPK27) in *C. pepo* were confirmed to have more than five EF-hand domains via both online tools ScanProsite and SMART. More detailed information, for example for myristoylation and palmitoylation sites, is also provided in Table S2.

### 2.2. Structure Variation Analysis of CpCDPK19 and CpCDPK27

**Figure 1:**
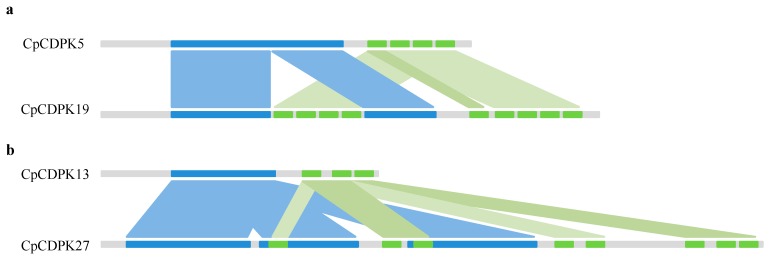
Gene structure variation analysis of CpCDPK19 and CpCDPK27. Syntenic analysis of CpCDPK5 and CpCDPK19 (**a**), as well as CpCDPK12 and CpCDPK27 (**b**). Blue and green rectangles represent STKs_CAMK protein kinase and EF-hand domain, respectively.

As mentioned above, CpCDPK19 and CpCDPK27 in *C. pepo* harbored nine and eight EF-hand motifs, respectively. Previous research indicates that *Cucurbita*, containing two sub-genomes A and B, originated from two progenitors that diverged from one another approximately 30.75 million years ago (Mya) [26]. Sequence analysis showed that CpCDPK5 and CpCDPK13, sharing the highest similarity with CpCDPK19 (amino acid identity: 93.74%) and CpCDPK27 (amino acid identity: 95.84%), contained four and three EF-domains, respectively (Table S2). Dot plot analysis indicated that the EF-hand domains were duplicated in CpCDPK19 compared to CpCDPK5, while both STKs_CAMK protein kinase and EF-hand domains were repeated in CpCDPK27 in contrast to CpCDPK13 (Figure S1). Based on the synteny analysis (Figure 1a), we inferred that a fragment containing four EF-hand motifs in CpCDPK19 was entirely duplicated and inserted into the STKs_CAMK protein kinase domain, and the fifth EF-hand motif resulted from another duplication event. Compared to ClCDPK13, ClCDPK27 harbored three copies of STKs_CAMK protein kinase domain. Moreover, the first EF-hand domain located in the second STKs_CAMK protein kinase, as well as the second and fourth EF-hand domains, were originated from the sixth EF-hand domain, while the third (existed in the third STKs_CAMK protein kinase) and the fifth EF-hand domains were two copies of the seventh motif (Figure 1b). Using the Cucurbit Genomics Database (CuGenDB) [29], both two genes CpCDPK19 and CpCDPK27 could be detected in the fruits of two different materials at transcriptional level (Figure S2). In summary, self-duplication of conserved domains may contribute to the gene structure variations, and result in sub-functionalization or neo-functionalization.

### 2.3. Construction of an Integrated Map for CDPK and CRK Genes

As the genomic distribution of *CDPKs* and *CRKs* in *Cucumis* [9,11], all identified genes in this study were also unevenly distributed on chromosomes in five species, with a few chromosomes harboring none of them (Figure S3). A recent study has confirmed that there are twenty chromosomes in *Cucurbita* species and nineteen of them could be divided into two sub-genomes A and B, except for chromosome 04, consisting of two segments from sub-genome A and one from sub-genome B [26]. Interestingly, almost equal numbers of *CDPKs* and *CRKs* were found to be retained in two sub-genomes (Table S3), consistent with the similar evolved ratios of gene loss or gain of two sub-genomes after polyploidization [26].

**Figure 2:**
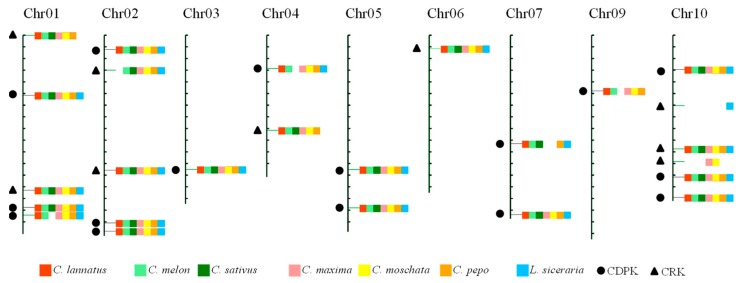
An integrated map of *CDPK* and *CRK* loci in Cucurbitaceae. All *CDPK* and *CRK* genes in Cucurbitaceae were mapped onto 11 chromosomes of watermelon 97103. Loci from *C. lannatus* (red), *C. melon* (light green), *C. sativus* (dark green), *C. maxima* (pink), *C. moschata* (yellow), *C. pepo* (dark yellow), and *L. siceraria* (blue) have been marked with different colors, as indicated in brackets. Black dots and triangles represent *CDPK* and *CRK* loci, respectively.

The evolutionary scenario of Cucurbitaceae paleohistory hypothesizes that the modern chromosomal structures in cucurbits were derived from an ancestral Cucurbitaceae karyotype (ACK) that consisted of 12 protochromosomes and experienced different times of chromosomal fission and fusion events [27]. Hence, to investigate the conserved loci of *CDPK* and *CRK* in Cucurbitaceae, an integrated map was constructed using watermelon chromosomes as reference, including 16 *CDPK* and nine *CRK* loci (Figure 2). Of these, nine loci (four *CDPK* and five *CRK*) exhibited Presence/Absence polymorphism among genomes, while the remaining 16 loci (12 *CDPK* and four *CRK*) were conserved and shared by all seven species (Figure 2 and Table S4). For example, the first locus on chromosome 01 was lost in *L. siceraria*, while the second locus on chromosome 02 was only absent in watermelon genome.

### 2.4. Phylogenetic Analysis of CDPK and CRK Gene Families

#### 2.4.1. In the Benincaseae Tribe

In the Benincaseae tribe, watermelon was predicted to diverge from its sister lineage bottle gourd around 10.4–14.6 million years ago (Mya) and from *Cucumis* 17.3–24.3 Mya [27]. To deeply analyze the phylogentic relationships of *CDPK* and *CRK* gene families in these four species, all full-length protein sequences of watermelon (18 CDPKs and six CRKs), melon (18 CDPKs and seven CRKs), cucumber (17 CDPKs and seven CRKs), and bottle gourd (17 CDPKs and seven CRKs) were aligned using the software MUSCLE, and were then used to construct an evolutionary tree using MEGA6.0 (Figure S4). Four CDPK groups (CDPK I, CDPK II, CDPK III, and CDPK IV) and one CRK group (CRK I) were observed in the distance tree. Combined with results from the integrated map, all groups (except for CDPK IV) were found to constitute homologs from at least four loci. Furthermore, most loci contained only one homolog from each individual species. Apparently, homologs from watermelon and bottle gourd were preferentially clustered together, compared to those from *Cucumis* species.

#### 2.4.2. In the Cucurbiteae Tribe

It has been reported that a whole genome duplication (WGD) event has occurred in the progenitor of the *Cucurbita* genus [26,27]. As shown in the phylogenetic tree constructed with all identified full-length protein sequences from *C. moschata* (30 CDPKs and 12 CRKs), *C. maxima* (31 CDPKs and 12 CRKs), and *C. pepo* (32 CDPKs and 12 CRKs), the majority of loci in five groups contained two copies of the homolog gene from each species (Figure S5). Generally, CDPKs or CRKs from the same sub-genome (A or B) of *C. moschata* and *C. maxima* grouped together in the distance tree. For example, both CmoCDPK7 and CmaCDPK8 from sub-genome B of two species clustered together in CDPK IV, while CmoCDPK14 and CmaCDPK15 from sub-genome A formed another sister clade. Based on these observations, origins of CDPKs on chromosome 4 in two *Cucurbita* species could be uncovered, although the boundary sites of three segments (two from sub-genome A and one from B) of chromosome 04 were ambiguous [26], e.g., CmoCDPK26 and CmaCDPK27 in Locus 15 (group CDPK I) were from sub-genome A (Table S3 and Figure S5), suggesting that the members (CmoCDPK9 and CmaCDPK10) in its sister clade originated from sub-genome B. Similarly, CmoCDPK8 and CmaCDPK9 were inferred to originate form sub-genome A, which formed the Locus 20 in group CDPK II. It is worthy to note that both loci 06 and 24 formed two independent clades in the phylogenetic tree, due to having far more CDPK members.

#### 2.4.3. In the Cucurbitaceae Family

**Figure 3:**
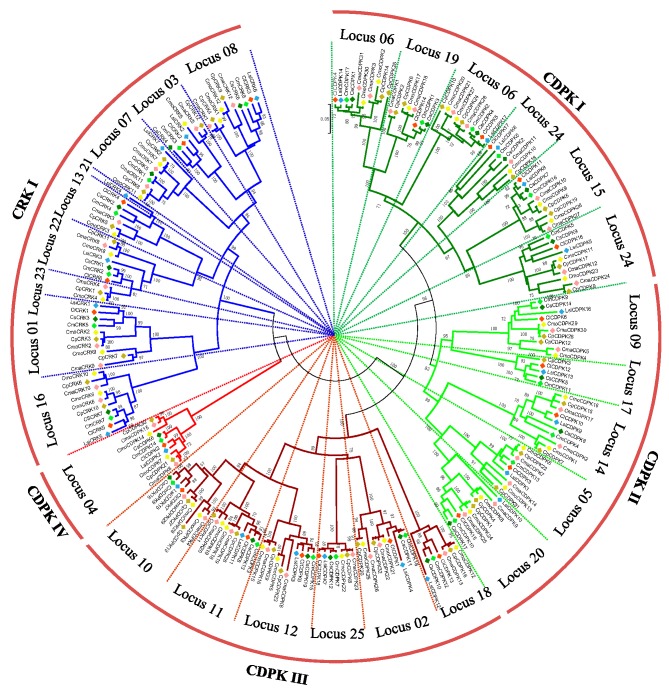
Phylogenetic tree of *CDPK* and *CRK* genes from Cucurbitaceae species. Four CDPK groups and one CRK group can be found in the tree, which were further divided into 25 loci. Numbers on nodes represent bootstrap values, and values <65 are not shown.

To deeply analyze the evolutionary history of *CDPK* and *CRK* gene families in Cucurbitaceae, a total of 226 protein sequences were used to construct a phylogenetic tree (Figure 3). Similar to topological structures mentioned above, four CDPK and one CRK group could be further divided into 25 loci. Notably, six homologs (LsiCRK1, CsCDPK3, CsCDPK19, CpCRK1, CpCDPK1, and CpCDPK2), which failed to be mapped on the integrated map due to lack of enough flanking sequences, were clustered with their orthologs in the phylogenetic trees. CDPKs or CRKs from watermelon and bottle gourd still cluster together in most loci. Compared to sub-genome A, members from sub-genome B usually gathered together with those from the Benincaseae tribe, except for two loci in group CRK I (Figure S6), which is consistent with the evolutionary scenario of modern Cucurbitaceae genomes [27]. CDPK IV, as the smallest group in CDPK lineage, was clustered with CRK I rather than the other three CDPK groups in phylogenetic trees, indicating that groups CDPK IV and CRK I may have originated from a common ancestor [10,11,30].

For the comparative evolutionary analysis of two gene families in plants, a phylogenetic tree was constructed using the 226 protein sequences in Cucurbitaceae, as well as that from *Arabidopsis* (34 CDPKs and eight CRKs, Cruciferae), tomato (29 CDPKs and six CRKs, Solanaceae), pepper (31 CDPKs and five CRKs, Solanaceae), and rice (31 CDPKs and five CRKs, Poaceae) (Figure S7). There is no doubt that all groups contained homologs from these four families, suggesting that both gene families have conserved basal architectures in their evolutionary process.

### 2.5. Exon-Intron Organization in CDPK and CRK Groups

The exon-intron organization, as well as the intron numbers, can also provide important evidence to analyze the evolutionary history within gene families [9,11,30]. To obtain further insight into the phylogenetic relationships of two gene families in Cucurbitaceae, gene structures of all 226 CDPKs and CRKs were comparatively depicted, dependent on their gene annotation profiles and genomic sequences (Figure S8). The majority of members in group CDPK I contained six introns with a distinct intron phase pattern 111000, while most CDPKs in group CDPK II had seven introns, sharing a similar intron pattern of 1110020. CDPK III, as a peripheral sister clade of groups CDPK I and II, contained two major intron phases. For instance, 13 out of 17 CDPKs in loci 02 and 25 shared an intron phase 111000, which is identical to that of group CDPK I, while 28 of 37 CDPKs in the other four loci had an intron pattern of 0111000 (Figure S8). CDPK IV, as the smallest group, contained only 10 members with 11 or 12 introns. Among of them, eight CDPKs were constituted of 11 introns with a phase pattern of 02201010000, while the remaining two genes (*CsCDPK6* and *CmoCDPK7*) had 12 introns with an extra intron gain at the 5’ or 3’ end. In group CRK I, 41 out of 63 members contained 10 introns with a phase pattern 0220110000, showing high similarity with that in group CDPK IV. Notably, members in the same loci usually had similar exon or intron lengths, such as Locus 25 in group CDPK III.

### 2.6. Duplication and Syntenic Analysis of CDPK and CRK Gene Families

In addition to whole genome duplication (WGD) events, both tandem and segmental duplications have also been reported to play vital roles in the expansion and function of a gene family [11,31,32]. To further explore the possible evolutionary relationships of *CDPK* and *CRK* gene families in Cucurbitaceae, duplication events were investigated in six species (including watermelon, cucumber, bottle gourd, and three *Cucurbita* species). Similar to the melon genome [11], only one or two segmental duplication events were detected in three Benincaceae genomes, with syntenic regions no more than 3.0 Mb (Table S5). However, many more segmental duplication events were observed in *Cucurbita* genus, most of which occurred between sub-genomes.

**Figure 4:**
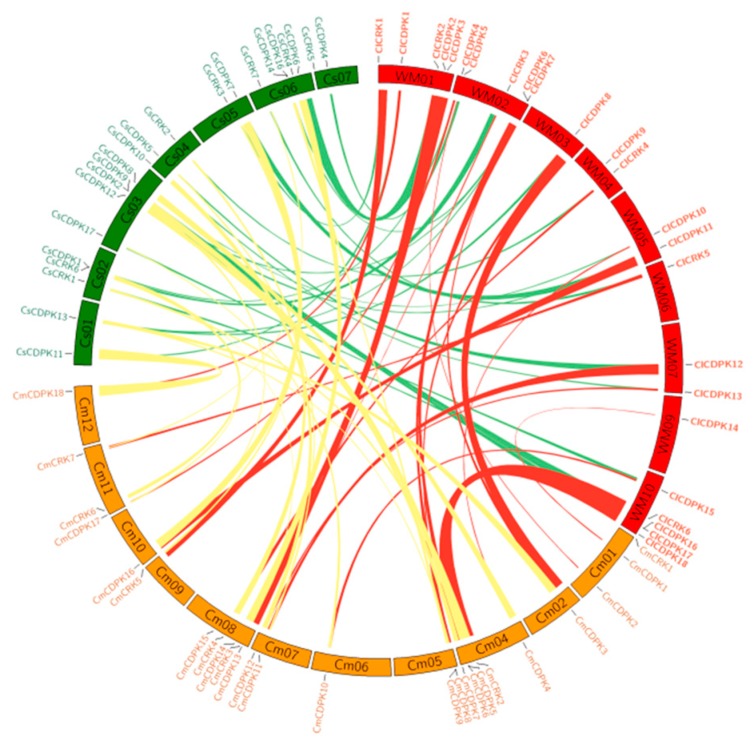
Synteny analysis of *CDPK* and *CRK* genes among watermelon, cucumber, and melon. Chromosomes of three species (watermelon, cucumber, and melon) were depicted in different colors (red, green, and yellow) and in circle form. The approximate distributions of each *CDPK* and *CRK* are presented by short black lines on the circle. Colored curves denote the details of syntenic regions containing *CDPK* and *CRK* genes among genomes.

In the Benincaseae tribe, watermelon, melon, and cucumber are important cucurbit crops widely cultivated throughout the world. Synteny analyses revealed that similar numbers of syntenic regions were detected among three genomes, with average fragment lengths not exceeding 2.5 Mb (Figure 4 and Table S6). As a close sister lineage of *Citrullus*, approximately 15 collinear regions were found between watermelon and bottle gourd, with the largest one spanning about 16.6 Mb. In the Cucurbiteae tribe, many more collinear regions were detected, with the largest one (21.3 Mb) existing between *C. moschata* and *C. pepo* (Table S6).

### 2.7. Expression Profiles of ClCDPK and ClCRK Genes in Different Tissues

**Figure 5:**
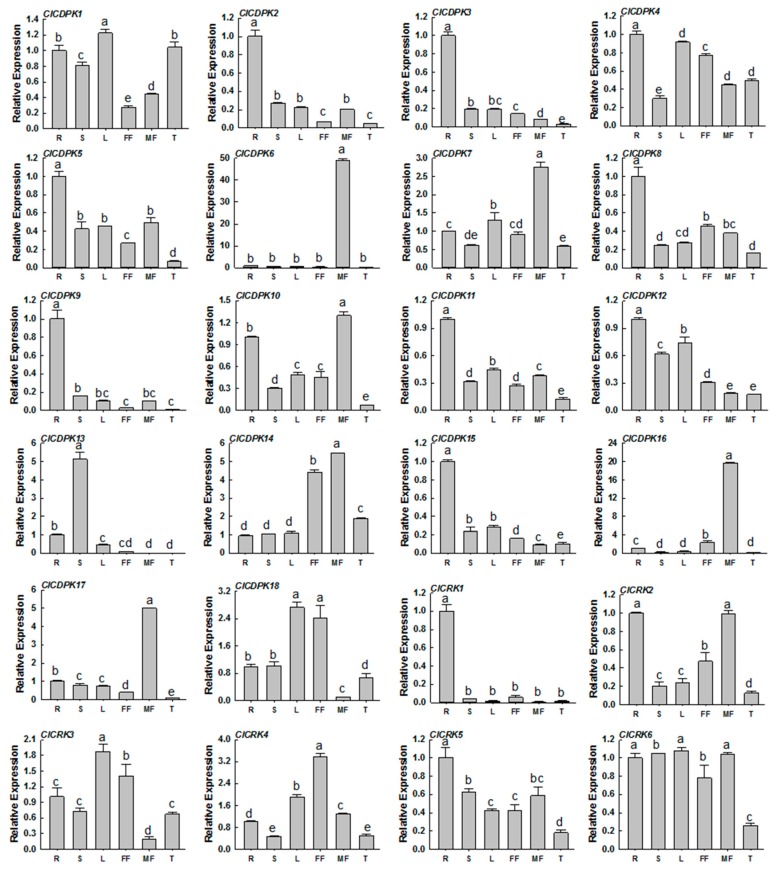
Expression profiles of *ClCDPK* and *ClCRK* genes in different tissues. The transcript levels of the respective genes in roots were used as references and set to a value of 1. The data were showed as means value ± SD. All experiments were performed with three independent replicates. R = roots; S = stems; L = leave; FF = female flowers; MF = male flowers; T = tendrils.

To assess the potential functions of *ClCDPK* and *ClCRK* genes, their expression patterns were investigated in six different tissues, including roots, stems, leaves, tendrils, male flowers, and female flowers. As shown in Figure 5, all identified *ClCDPKs* and *ClCRKs* could be detected in at least one tissue. Some *ClCDPKs* and *ClCRKs*, such as *ClCDPK2*, *ClCDPK3*, *ClCDPK9*, *ClCDPK15*, and *ClCRK1*, showed significantly elevated expression levels in the root, while *ClCDPK6*, *ClCDPK16*, and *ClCDPK17* were strongly expressed in the male flower. Interestingly, *CmCDPK6* in melon, the ortholog of *ClCDPK17* in locus 24, was also reported to have a high transcriptional abundance in the male flower [11]. Moreover, orthologs of *ClCDPK6* and *ClCDPK16* in melon (*CmCDPK9* and *CmCDPK5*) and cucumber (*CsCDPK14* and *CsCDPK9*) were also confirmed to retain high accumulations in male flowers [9,11].

### 2.8. Expression Patterns of ClCDPK and ClCRK Genes under Abiotic Stresses

**Figure 6:**
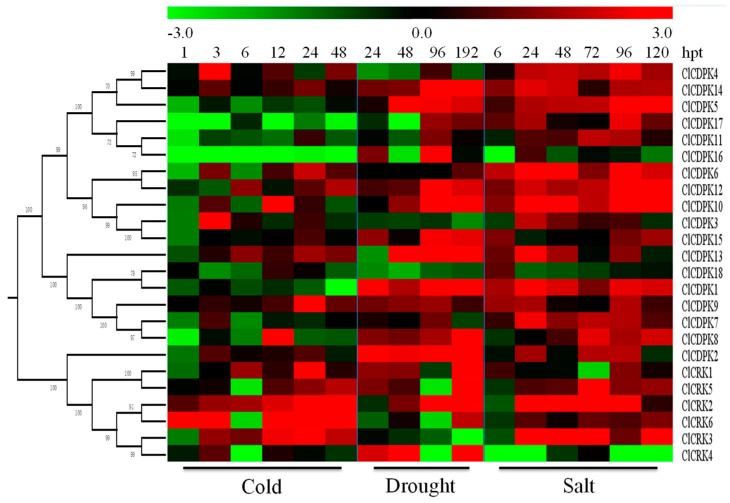
Expression patterns of *ClCDPK* and *ClCRK* genes under abiotic stresses. The abiotic stresses are displayed at the low end. The relative transcript level was log_2_ transformed and visualized as a heat map via Mev4.8.1, using red to indicate increased expression level and green to indicate decreased expression level.

Accumulation studies showed that *CDPK* and *CRK* genes are widely involved in the adaptations to environmental stimuli, and that their expression levels are affected by drought, salt, and cold [2,9,11]. To investigate the potential roles of *ClCDPKs* and *ClCRKs* in response to abiotic stresses, their dynamic expressions were analyzed under drought, salt, and cold treatments (Figure 6). Compared to cold stimuli, far more *ClCDPKs* and *ClCRKs* could be induced by drought and NaCl treatments, and seven genes could be up-regulated by both drought and NaCl treatments, including *ClCDPK1*, *ClCDPK5*, *ClCDPK6*, *ClCDPK9*, *ClCDPK10*, *ClCDPK12*, and *ClCDPK14*. Following cold treatment, the transcription levels of four genes (*ClCDPK1*, *ClCDPK5*, *ClCDPK16*, and *ClCDPK17*) were down-regulated, while gene *ClCRK2* was continuously up-regulated at all treatment times. Compared to *ClCDPK3* and *ClCDPK18* down-regulated by drought stress, many more genes were obviously up-regulated, such as *ClCDPK1*, *ClCDPK2*, *ClCDPK8*, *ClCDPK9*, *ClCDPK12*, and *ClCDPK14*. In response to NaCl stress, the majority of *ClCDPK* and *ClCRK* genes were up-regulated, with few exceptions (Figure 6).

### 2.9. Expression Patterns of ClCDPK and ClCRK Genes under Hormone Treatments

Previous studies have indicated that *CDPKs* and *CRKs* are involved in the signaling pathways of various plant hormones [19,33]. Here, expression profiles of *ClCDPKs* and *ClCRKs* were investigated in response to four plant hormones ABA, SA, MeJA, and ETH.

**Figure 7:**
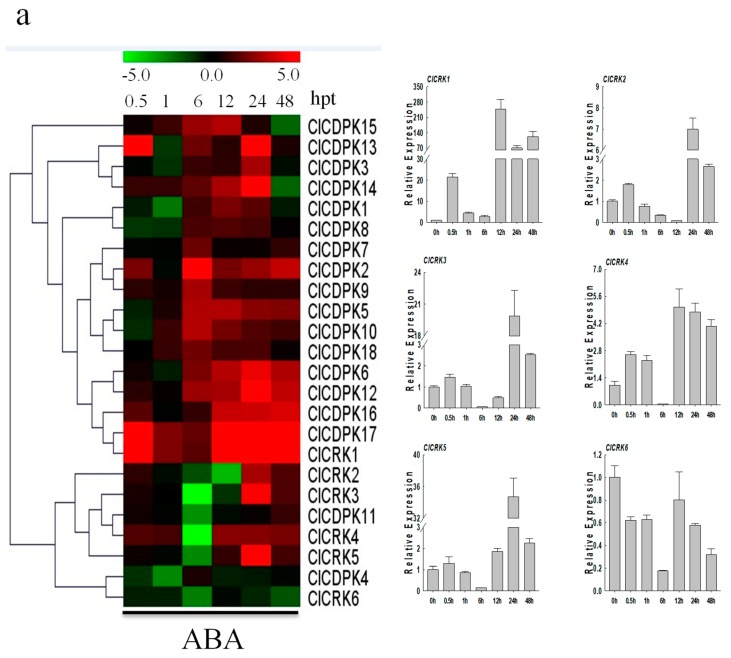

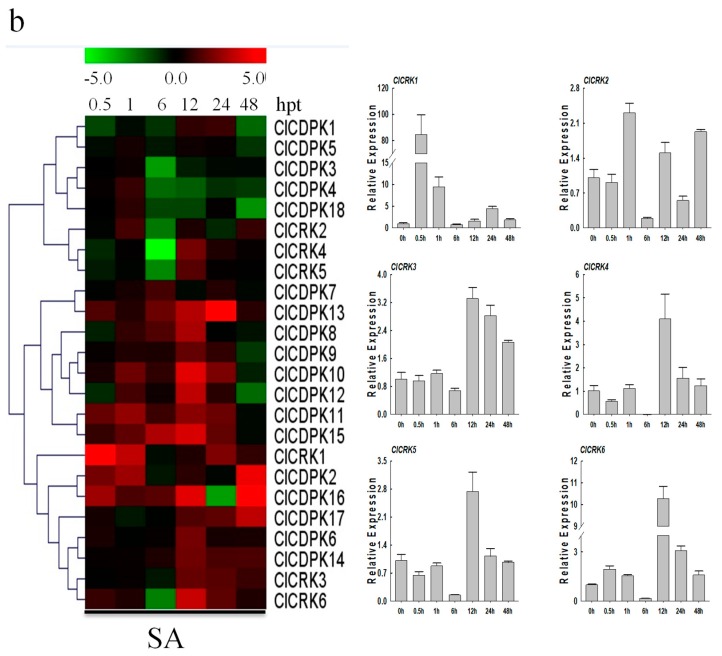
Expression patterns of *ClCDPK* and *ClCRK* genes under ABA and SA hormone treatments. (**a**) Expression levels of *ClCDPKs* and *ClCRKs* under ABA stress visualized as a heat map (Left). Detailed expression patterns of *ClCRKs* under ABA stress (Right). (**b**) Expressions of *ClCDPKs* and *ClCRKs* under SA stress visualized as a heat map (Left). Detailed expression patterns of *ClCRKs* under SA stress (Right). The relative transcript level was log_2_ transformed and visualized as a heat map via Mev4.8.1, using red to indicate increased expression level and green to indicate decreased expression level.

**Figure 8:**
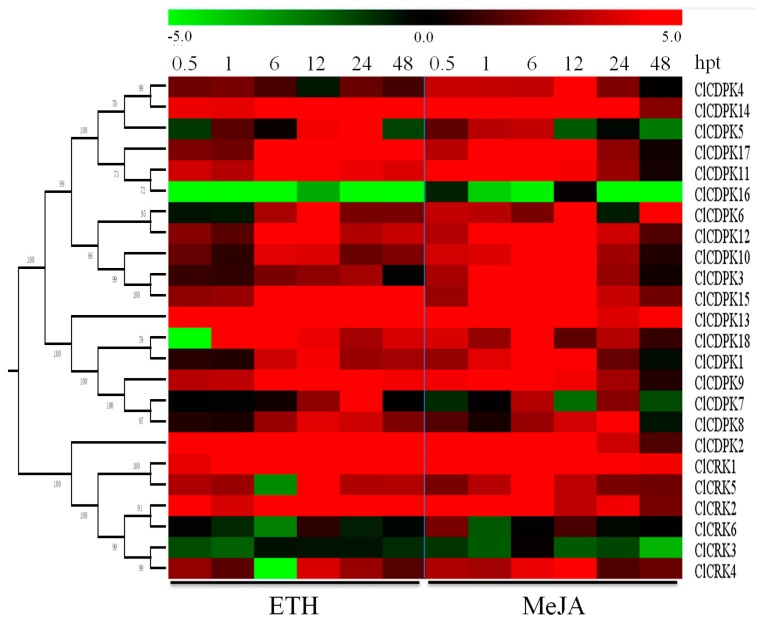
Expression patterns of *ClCDPK* and *ClCRK* genes under ETH and MeJA hormone stresses. The abiotic stresses are indicated at the low end. The relative transcript level was log_2_ transformed and visualized as a heat map by Mev4.8.1, using red to indicate increased expression level and green to indicate decreased expression level.

Increasing evidence has shown that *CDPK* and *CRK* genes could participate in ABA-mediated signal transduction in plants [17,34,35]. In the present study, the majority of *ClCDPKs* were found to be induced by ABA treatment, with transcript abundance retaining at higher levels from 6 to 24 hpt (Figure 7a). Interestingly, all *ClCRKs* except for *ClCRK1*, were sharply down-regulated at 6 hpt, which is similar to observations in melon [11]. Following SA treatment, expression levels of most *ClCDPKs* and *ClCRKs* remained either unchanged or slightly changed at 0.5 and 1 hpt (Figure 7b). However, SA application significantly induced or reduced their transcript levels at 6 or 12 hpt. Notably, *ClCRKs* were also found to have decreased at 6 hpt, which is similar to the tendency mentioned above. In contrast to the responses to ABA and SA treatments, almost all *ClCDPKs* and *ClCRKs* showed continuous over-expression in response to ETH and MeJA stimuli, except for genes *ClCDPK16* and *ClCRK3* (Figure 8). Moreover, transcript abundances of most *ClCDPKs* and *ClCRKs* had either sharply increased or decreased at 0.5 hpt, implying their rapid response to ETH and MeJA stimuli. In summary, these expression analyses indicated that *ClCDPKs* and *ClCRKs* could be involved in the regulatory pathways of plant hormones, and thus participate in the plant defense against environmental stresses.

## 3. Discussion

### 3.1. Characteristic Features of CDPK and CRK Genes in Cucurbitaceae

Functioning as both Ca^2+^ sensors and effectors, the *CDPK* gene family has been identified throughout the plant kingdom, as well as in several protozoa, but are absent in animals [4,28]. For instance, 34 *CDPKs* and eight *CRKs* were identified genome-wide in *Arabidopsis* [13], 29 *CDPKs* and six *CRKs* in tomato [10], 31 *CDPKs* and five *CRKs* in pepper [15], 30 *CDPKs* and nine *CRKs* in poplar [36], and 31 *CDPKs* and five *CRKs* in rice [12,14]. Moreover, approximately 19, 41, and 40 *CDKPs* have been detected in the genomes of grape [19], cotton [30], and maize [37], respectively. In Cucurbitaceae, 18 *CDPKs* and seven *CRKs* have been found in the melon genome [11], while 19 *CDPKs* were identified in cucumber by Xu et al. [9]. In this study, a total of 128 *CDPK* and 56 *CRK* genes were identified in six Cucurbitaceae species, including *C. lanatus*, *C. sativus*, *C. moschata*, *C. maxima*, *C. pepo*, and *L. siceraria* (Table 1). The numbers of *CDPKs* and *CRKs* are much higher in three *Cucurbita* species than in four Benincaseae tribe species (*C. lanatus*, *C. melon*, *C. sativus*, and *L. siceraria*), which may be due to a WGD event during the origin of this genus [25,26]. Moreover, the numbers of *CRKs* in three *Cucurbita* genomes are much higher than in other species, such as tomato, pepper, and rice, although these species contain similar copies of *CDPKs*.

Tandem, segmental, and whole genome duplication events are confirmed to play important roles in the expansion of gene families. Approximately 12, 13, and seven segmental duplications were reported to exist in poplar, cotton, and rice genomes [14,30,36], while many more events were found in three *Cucurbita* species that mainly occurred between two sub-genomes (Table S5). However, only a few segmental duplication events (one or two) were detected in genomes of watermelon, cucumber, bottle gourd, and melon [11], which may cause the low copy numbers of *CDPK* and *CRK* genes. In the present study, the majority of CDPKs contained four EF-hand motifs (Table S2), which is consistent with observations found in other species [7,11,19]. However, two CDPKs (CpCDPK19 and CpCDPK27) in *C. pepo* with detectable transcriptional levels had been confirmed to contain nine and eight EF-hands, respectively. Dot plot analysis showed that self-duplications of the STKs_CAMK protein kinase or EF-hand domains resulted in the gene structure variations (Figure 1), which may affect their functional specificity.

### 3.2. Conserved Evolution of CDPK and CRK Genes in Cucurbitaceae

Generally, *CDPK* and *CRK* genes are randomly distributed in genomes [10,11,15,36], which has also been validated in Cucurbitaceae species (Figure S3). Using watermelon chromosomes as reference, an integrated map was obtained and contained 16 *CDPK* and nine *CRK* loci, harboring almost all *CDPK* and *CRK* genes identified in Cucurbitaceae (Figure 2 and Table S4). Of these, nine loci (four *CDPK* and five *CRK*) exhibited Presence/Absence polymorphisms, while the remaining 16 loci (12 *CDPK* and four *CRK*) were shared by all seven species, implying that the flanking regions of most *CDPK* and *CRK* genes were conserved in these species during the evolutionary process.

Increasing evidence indicates that topological structures of *CDPK* and *CRK* gene families are conserved, with four *CDPK* and one *CRK* groups in phylogenetic trees [10,11,28,36]. In the present study, group CDPK IV was found to be close to CRK I rather than the other three CDPK groups in distance trees (Figure 3 and Figures S4, S5 and S7), confirming that CDPK IV and CRK I may originate from a common ancestor [10,11,30]. Moreover, the five groups can be further divided into 25 loci, according to the integrated map. The evolutionary scenario of seven modern Cucurbitaceae species revealed that watermelon diverged from bottle gourd around 10.4–14.6 Mya and from *Cucumis* 17.3–24.3 Mya; however, the progenitor B of *Cucurbita* diverged from Benincaseae around 25.5–27.0 Mya, and progenitor A diverged from the common ancestor of progenitor B and Benincaseae around 29.9–31.6 Mya [26,27]. In agreement with this evolutionary scenario, orthologs from watermelon and bottle gourd usually gathered together in phylogenetic trees, and genes from sub-genome B were preferentially clustered with those from Benincaseae tribe genomes (Figure 3 and Figure S7).

Both exon-intron structures and intron numbers can reflect the evolution, expansion, and functional relationships within a gene family, which were caused by three main types of mechanisms, including exon/intron gain/loss, exonization/pseudoexonization, and insertion/deletion [11,19,38]. Exon-intron organization analyses revealed that each group contained one or two major intron phase patterns (Figure S8). For instance, the majority of homologs in group CDPK I contained six introns with a distinct intron phase pattern of 111000, while most members in CDPK II had seven introns sharing a similar intron phase 1110020. As a peripheral sister clade of CDPK I and II, CDPK III contained two major intron phases and one of them was identical to that of group CDPK I. Moreover, the major intron pattern (02201010000) of eight members in CDPK IV is similar to that (0220110000) of most *CRK* homologs in CRK I (Figure S8). Combined with the topological structures of CDPK IV and CRK I, we infer that group CDPK IV is the ancient lineage of CDPK gene family, which may have diverged from the last common ancestor with CRK I before the divergence of monocots and dicots [28]. 

### 3.3. Functional Comparison of CDPK and CRK Genes

*CDPKs* and *CRKs* have been confirmed to play crucial roles in the signal pathways in response to various environmental stresses [4,5,28]. The systemic expression profiles of *CDPK* and *CRK* genes in *Cucumis* species under different stimuli have been reported recently [9,11]. Consequently, dynamic expression levels of *CDPKs* and *CRKs* under different stimuli were investigated in detail in watermelon. Compared to cold stimuli, far more *ClCDPKs* and *ClCRKs* were induced under drought and NaCl treatments (Figure 6), which differed from the expression trends of most homologs that were up-regulated by cold stress in cucumber and melon [9,11]. In watermelon, expression levels of *ClCDPK14* were up-regulated by all three stresses (cold, drought, and salt); its ortholog *CmCDPK1* in melon, could be also induced by cold and salinity treatments [11]. Completely different expression trends were also observed among species. For example, both genes *ClCDPK6* and *ClCDPK12*, with a close relationship in group CDPK II, could be induced by salt stress (Figure S4 and Figure 6), while expression levels of their orthologs in melon (*CmCDPK9* and *CmCDPK11*) and cucumber (*CsCDPK14* and *CsCDPK5*) exhibited contrary responses to salinity stimuli [9,11]. Similarly, transcriptional levels of *ClCRK2* and its paralog *ClCRK3*, as well as their orthologs *CmCRK3* and *CmCRK6* in melon, were increased under low temperature; however, these two groups of genes showed different expression trends under salt stress between watermelon and melon. Taken together, we inferred that the functional fates of some orthologs may be diversified during species evolution. The phytohormone ABA has been reported to be widely involved in the response of plants to biotic and abiotic stresses [17,34,35]. For instance, AtCPK4 and AtCPK11 in *Arabidopsis* can positively mediate the CDPK/calcium-mediated ABA signaling pathways via phosphorylation of two ABA-responsive transcription factors, ABF1 and ABF4, and loss-of-function mutations decrease the tolerance of seedlings to salt stress [17]. As their closest homolog in the phylogenetic tree (Figure S7), transcript abundance of *ClCDPK14* was also up-regulated under ABA and salt stimuli (Figure 6 and Figure 7). The biosynthesis of ABA could be induced by drought, leading to stomatal closure [17,39]. In *Arabidopsis*, the AtCPK4 and AtCPK11 are partially involved in ABA-induced stomatal closure, and the double mutant lost more water from leaves compared to single mutants [17]; gene *ClCDPK14*, as their closed homolog in watermelon, was also activated by drought (Figure 6). Additionally, gene *AtCPK6* has been proven to be involve in the response to drought and salt stress as a positive regulator [40]. Intriguingly, *ClCDPK5* in watermelon, sharing the highest sequence similarity with *AtCPK6*, was also up-regulated by drought (or salt) and ABA stresses, indicating that they might function in similar pathways. In this study, the transcription levels of most *ClCDPKs* were increased by exogenous ABA (Figure 7), similar to the expression trend in grape but different to that in *Cucumis* species [9,11,19].

In watermelon, transcription levels of four genes (*ClCDPK1*, *ClCDPK5*, *ClCDPK16*, and *ClCDPK17*) were down-regulated by cold treatment, while only one gene (*ClCRK2*) was continuously up-regulated at all treatment times (Figure 6). Moreover, complex expression patterns were also observed under cold stimuli, such as *ClCRK5* and *ClCRK6*. Three up-regulated genes (*ClCDPK1*, *ClCDPK2*, and *ClCDPK5*) and one down-regulated gene (*ClCDPK18*) under continuous drought treatment in this study have also been detected and regarded as different expression genes in our previous study [41]. Following salt treatment, gene *ClCDPK6* was significantly up-regulated at all treatment times, which is similar to its ortholog *CsCDPK14* in cucumber but in contrast with its ortholog *CmCDPK9* in melon [9,11]. Additionally, two pairs of segmental duplications were detected in watermelon: *ClCDPK7*/*ClCDPK8* and *ClCDPK6*/*ClCDPK16* (Table S5). Interestingly, *ClCDPK7* and *ClCDPK8* had similar expression patterns under most treatments, while expression tendency of *ClCDPK6* was usually opposite to *ClCDPK16* (Figure 6, Figure 7 and Figure 8), inferring that genes *ClCDPK6* and *ClCDPK16* may have undergone sub-functionalization after duplication.

Generally, *CDPK* genes are ubiquitously expressed in plant organs, with some showing organ- or tissue-specific expression [19,21]. In the present study, most identified *ClCDPKs* and *ClCRKs* could be detected in at least one tissue, with at least five genes showing extremely high expression levels in specific organs (Figure 5). For instance, *ClCDPK17* in locus 24 showed a high expression level in the male flower, similar to its ortholog *CmCDPK6* in melon, which preferentially accumulated in male flowers [11]. In *Arabidopsis*, both genes *AtCPK17* and *AtCPK20* are reported to be preferentially expressed in mature pollen to regulate the growth of pollen tubes [21,42]. Interestingly, their phylogenetically-close homologs *ClCDPK6* and *ClCDPK16* in watermelon, as well as that in melon (*CmCDPK9* and *CmCDPK5*) and cucumber (*CsCDPK14* and *CsCDPK9*) (Figure S7), were also detected with high transcriptional abundances in male organs [9,11], indicating their conserved and important roles in the development of male flowers. 

## 4. Materials and Methods

### 4.1. Identification and Biochemical Characterization of CDPKs and CRKs

For genome-wide identification of *CDPK* and *CRK* genes in Cucurbitaceae species, protein sequences of 18 CmCDPKs, seven CmCRKs, and 19 CsCDPKs were obtained according to recently published studies [9,11]. Then, using BLASTp program with an E-value setting of 1.0 × 10^−5^, these protein sequences were used as queries to search against the predicted protein files of watermelon (*C. lanatus*, v1), cucumber (*C. sativus*, v1), *Cucurbita* genus (*C. moschata,* v1; *C. maxima,* v1.1; *C. pepo*, v4.1), and bottle gourd (*L. siceraria*, v1), which were downloaded from the Cucurbit Genomics Database (http://cucurbitgenomics.org/). Additionally, Hidden Markov Model (HMM) profiles of the core protein kinase domain (PF00069) and EF-hand_7 domain (PF13499) were downloaded from the Pfam database (http://pfam.xfam.org/), and were also subjected to searches for *CDPKs* and *CRKs* with software HMMER 3.0 (default parameters). The reliability of candidates was verified through searching against the NCBI nr database, and all non-redundant putative genes were tested for the presence of core domains using ScanProsite (http://prosite.expasy.org/scanprosite/). Finally, all candidate genes were further characterized with the following online tools: ProtParam (http://web.expasy.org/protparam/), ScanProsite (http://prosite.expasy.org/scanprosite/), SMART (http://smart.embl-heidelberg.de/), ExPASy (http://web.expasy.org/myristoylator/), and CSS-plam 4.0 (http://csspalm.biocuckoo.org/). Dot plot analysis was performed using the software Geneious (http://www.geneious.com). 

### 4.2. Chromosomal Location of CDPK and CRK Genes

The genomic distributions of *CDPKs* and *CRKs* on chromosomes were drawn using the software TBtools (http://cj-chen.github.io/tbtools/). To construct an integrated map, all identified CDPKs and CRKs, as well as those reported in recent studies [9,11], were mapped onto watermelon chromosomes based on the syntenic relationships of their flanking genes using BLASTp method, with a stricter E-value setting to 1.0 × 10^−10^. Then, in-house Perl scripts were used to parse the resulting files and to visualize the chromosomal locations of *CDPK* and *CRK* loci.

### 4.3. Phylogenetic, Gene Structure, and Syntenic Analyses of CDPKs and CRKs

Full-length CDPK and CRK protein sequences were aligned using the software Muscle [43], and then were used to construct phylogenetic trees via MEGA 6.0 using the neighbor-joining method with 1000 bootstrap replicates [11]. To perform gene structure analyses, genomic and cDNA sequences of *CDPKs* and *CRKs* were obtained from their corresponding genomes. Then, the exon-intron organization was displayed via the online tool GSDS 2.0 (http://gsds.cbi.pku.edu.cn/). The destination tabular (-m 8) files of the BLASTp program (with an E-value setting of 1.0 × 10^−10^) and GFF profiles served as input documents for MCScanX to analyze the synteny relationships [44], which were then visualized using software CIRCOS (http://circos.ca/). 

### 4.4. Plant Material and Treatments

The watermelon inbred line “Y34” provided by the Cucurbits Germplasm Resource Research Group at the Northwest A&F University in China was used in this study. For tissue-specific analysis, germinated seeds of watermelon “Y34” were directly sown in the experimental base, and six organs (roots, stems, leaves, tendrils, and both male and female flowers) were independently sampled during the fruit maturation period (approximately 60–70 days after sowing). For stress experiments, seedlings were cultured in plastic pots (8 cm × 7 cm × 7 cm) filled with commercial peat-based compost (Shaanxi Yufeng Seed Industry Co., Ltd., Yangling, China). All plants were grown under springtime natural light with temperatures of 28–35 °C/16–20 °C (day/night) in a greenhouse, which were uniformly watered and nourished weekly with half-strength Hoagland’s solution before treatments. Four weeks after sowing, seedlings were used for the following treatments. 

For the salinity treatment, seedlings irrigated with 300 mM NaCl solution (80 mL per plant) were sampled at 6, 24, 48, 72, 96, and 120 h post-treatment (hpt), while plants irrigated with distilled water were used as control. Compared to seedlings planted in a growth chamber at 27 ± 1 °C and 80% humidity under a light intensity of 300 mmol·m^−2^·s^−1^ PPFD, leaves of plants kept at 4 °C were sampled at 1, 3, 6, 12, 24, and 48 hpt for cold treatment. To simulate a national drought treatment [45], all seedlings were uniformly well-watered to 70 ± 5% field capacity based on their weight. Then, leaves of drought-treated (unwatered) and control plants were sampled at 24, 48, 96, and 192 hpt. Leaves sprayed with 100 μM abscisic acid (ABA) [46], 1 mM salicylic acid (SA) [47], 100 μM methyljasmonate (MeJA), and 10 mM ethephon (ETH) [48] were collected at 0.5, 1, 6, 12, 24, and 48 hpt for hormone treatments, while control seedlings were only sprayed with equal volumes of the corresponding solution without hormones. In this study, leaves of four plants were pooled at each time point for each treatment with three biological replicates, which were immediately frozen in liquid nitrogen and stored at −80 °C until further analysis.

### 4.5. RNA Isolation and qRT-PCR

The total RNA of samples was extracted using the RNASimple Total RNA Kit (TIANGEN, China) following the manufacturer’s instructions. Then, approximately 1 μg of total RNA was used to synthesize the first strand of cDNA using the FastKing RT Kit with gDNase (TIANGEN, China). Gene-specific primers for *ClCDPKs* and *ClCRKs* are listed in Table S1. Amplification was conducted in a 20 μL reaction volume, containing 10.0 μLSYBR Green Premix, 0.8 μL of each primer (10 μM), and 1.0 μL cDNA template (80 ng/μL), which was diluted with ddH_2_O to 20 μL. The PCR conditions consisted of pre-denaturing at 95 °C for 5 min, followed by 40 cycles of 95 °C for 10 s and 60 °C for 30 s. The watermelon *β-actin* gene (*Cla007792*) was used as the internal control gene [49]. Each treatment was repeated thrice, and all data were calculated for relative expressions following the 2^−ΔΔCt^ method, as described by Livak and Schmittgen [50]. The relative expressions were then log2 transformed and visualized in a heat map using Mev 4.8.1 (http://www.mybiosoftware.com/). All data were analyzed via IBM SPSS Statistics 21 and values were presented as the means ± SD of three biological and three technical replicates (Table S7). The significance of expression between treatments and controls was evaluated by one-way ANOVA and Duncan’s multiple range tests.

## 5. Conclusions

In the present study, a total of 128 *CDPK* and 56 *CRK* genes were identified in six Cucurbitaceae species. Using the watermelon genome as reference, an integrated map containing 25 loci (16 *CDPK* and nine *CRK* loci) was obtained, 16 of which (12 *CDPK* and four *CRK*) were shared by all seven Cucurbitaceae species. Combined with exon-intron organizations, topological analyses indicated an ancient origination of groups CDPK IV and CRK, which will contribute to elucidating the evolutionary history of these two gene families in Cucurbitaceae. Moreover, expression patterns of *ClCDPKs* and *ClCRKs* under different abiotic stresses were also performed in this study. Importantly, comparative analyses indicated a subset of valuable orthologous genes for future functional characterizations. For instance, both *ClCDPK6* and its ortholog *CsCDPK14* in cucumber could be induced by salinity, while *ClCDPK6* and *ClCDPK16*, as well as their orthologs in *Cucumis*, maintained high expression levels in male flowers, which may play important roles in plant organ development and response to environmental stresses.

## Figures and Tables

**Table 1:** Numbers and characteristic properties of *CDPKs* and *CRKs* in Cucurbitaceae species.

Species	No. of Genes (CDPK/CRK)	No. of aa (CDPK/CRK)	MW (CDPK/CRK)	pI (CDPK/CRK)	Source
*C. lanatus*	18/6	491~662/550~696	55.23~74.32/61.88~77.97	5.09~8.84/6.84~9.17	In this study
*C. melon*	18/7	501~661/561~622	56.25~74.22/63.12~69.44	5.06~8.75/6.91~9.15	Zhang et al, 2017 [11]
*C. sativus*	17/7	501~661/556~726	56.30~74.30/62.24~81.23	4.99~8.99/7.20~9.33	Xu et al, 2015 [9]; In this study
*L. siceraria*	17/7	353~1094/555~636	39.92~125.03/62.25~70.80	5.14~8.74/6.82~9.18	In this study
*C. moschata*	30/12	318~820/517~758	36.26~90.89/57.54~84.44	5.02~9.10/6.49~9.31	In this study
*C. maxima*	31/12	499~1230/278~687	56.17~138.34/31.62~76.92	5.13~9.03/5.94~9.21	In this study
*C. pepo*	32/12	439~1393/342~609	49.44~157.82/39.21~68.01	4.84~8.49/5.97~9.28	In this study

## Supplementary Materials

Supplementary materials can be found at https://www.mdpi.com/1422-0067/20/10/2527/s1.

## Abbreviations

CDPK	Calcium-dependent protein kinase
CRK	CDPK-related kinases
hpt	hours post-treatment
ABA	abscisic acid
SA	salicylic acid
MeJA	methyljasmonate
ETH	ethephon

## References

[1] Hepler P.K. (2005). Calcium: A central regulator of plant growth and development. Plant Cell.

[2] Boudsocq M., Sheen J. (2013). CDPKs in immune and stress signaling. Trends Plant Sci..

[3] Ranty B., Aldon D., Galaud J.P. (2006). Plant calmodulins and calmodulin-related proteins: Multifaceted relays to decode calcium signals. Plant Signal. Behav..

[4] Hamel L.P., Sheen J., Seguin A. (2014). Ancient signals: Comparative genomics of green plant CDPKs. Trends Plant Sci..

[5] Ludwig A.A., Romeis T., Jones J.D. (2004). CDPK-mediated signalling pathways: Specificity and cross-talk. J. Exp. Bot..

[6] Boudsocq M., Willmann M.R., McCormack M., Lee H., Shan L., He P., Bush J., Cheng S.H., Sheen J. (2010). Differential innate immune signalling via Ca(2+) sensor protein kinases. Nature.

[7] Cheng S.H., Willmann M.R., Chen H.C., Sheen J. (2002). Calcium signaling through protein kinases. The Arabidopsis calcium-dependent protein kinase gene family. Plant Physiol..

[8] Liese A., Romeis T. (2013). Biochemical regulation of in vivo function of plant calcium-dependent protein kinases (CDPK). Biochim. Biophys. Acta.

[9] Xu X., Liu M., Lu L., He M., Qu W., Xu Q., Qi X., Chen X. (2015). Genome-wide analysis and expression of the calcium-dependent protein kinase gene family in cucumber. Mol. Genet. Genom. MGG.

[10] Wang J.P., Xu Y.P., Munyampundu J.P., Liu T.Y., Cai X.Z. (2016). Calcium-dependent protein kinase (CDPK) and CDPK-related kinase (CRK) gene families in tomato: Genome-wide identification and functional analyses in disease resistance. Mol. Genet. Genom. MGG.

[11] Zhang H., Wei C., Yang X., Chen H., Yang Y., Mo Y., Li H., Zhang Y., Ma J., Yang J. (2017). Genome-wide identification and expression analysis of calciumdependent protein kinase and its related kinase gene families in melon (Cucumis melo L.). PLoS ONE.

[12] Ray S., Agarwal P., Arora R., Kapoor S., Tyagi A.K. (2007). Expression analysis of calcium-dependent protein kinase gene family during reproductive development and abiotic stress conditions in rice (*Oryza sativa* L. ssp. indica). Mol. Genet. Genom. MGG.

[13] Hrabak E.M., Chan C.W., Gribskov M., Harper J.F., Choi J.H., Halford N., Kudla J., Luan S., Nimmo H.G., Sussman M.R. (2003). The Arabidopsis CDPK-SnRK superfamily of protein kinases. Plant Physiol..

[14] Asano T., Tanaka N., Yang G., Hayashi N., Komatsu S. (2005). Genome-wide identification of the rice calcium-dependent protein kinase and its closely related kinase gene families: Comprehensive analysis of the CDPKs gene family in rice. Plant Cell Physiol..

[15] Cai H., Cheng J., Yan Y., Xiao Z., Li J., Mou S., Qiu A., Lai Y., Guan D., He S. (2015). Genome-wide identification and expression analysis of calcium-dependent protein kinase and its closely related kinase genes in Capsicum annuum. Front. Plant Sci..

[16] Zhou L., Lan W., Jiang Y., Fang W., Luan S. (2014). A calcium-dependent protein kinase interacts with and activates a calcium channel to regulate pollen tube growth. Mol. Plant.

[17] Zhu S.Y., Yu X.C., Wang X.J., Zhao R., Li Y., Fan R.C., Shang Y., Du S.Y., Wang X.F., Wu F.Q. (2007). Two calcium-dependent protein kinases, CPK4 and CPK11, regulate abscisic acid signal transduction in Arabidopsis. Plant Cell.

[18] Ma S.Y., Wu W.H. (2007). AtCPK23 functions in Arabidopsis responses to drought and salt stresses. Plant Mol. Biol..

[19] Zhang K., Han Y.T., Zhao F.L., Hu Y., Gao Y.R., Ma Y.F., Zheng Y., Wang Y.J., Wen Y.Q. (2015). Genome-wide Identification and Expression Analysis of the CDPK Gene Family in Grape, Vitis spp.. BMC Plant Biol..

[20] Chen F., Fasoli M., Tornielli G.B., Dal Santo S., Pezzotti M., Zhang L., Cai B., Cheng Z.M. (2013). The evolutionary history and diverse physiological roles of the grapevine calcium-dependent protein kinase gene family. PLoS ONE.

[21] Myers C., Romanowsky S.M., Barron Y.D., Garg S., Azuse C.L., Curran A., Davis R.M., Hatton J., Harmon A.C., Harper J.F. (2009). Calcium-dependent protein kinases regulate polarized tip growth in pollen tubes. Plant J. Cell Mol. Biol..

[22] Huang S., Li R., Zhang Z., Li L., Gu X., Fan W., Lucas W.J., Wang X., Xie B., Ni P. (2009). The genome of the cucumber, Cucumis sativus L.. Nat. Genet..

[23] Garcia-Mas J., Benjak A., Sanseverino W., Bourgeois M., Mir G., Gonzalez V.M., Henaff E., Camara F., Cozzuto L., Lowy E. (2012). The genome of melon (Cucumis melo L.). Proc. Natl. Acad. Sci. USA.

[24] Guo S., Zhang J., Sun H., Salse J., Lucas W.J., Zhang H., Zheng Y., Mao L., Ren Y., Wang Z. (2013). The draft genome of watermelon (Citrullus lanatus) and resequencing of 20 diverse accessions. Nat. Genet..

[25] Montero-Pau J., Blanca J., Bombarely A., Ziarsolo P., Esteras C., Marti-Gomez C., Ferriol M., Gomez P., Jamilena M., Mueller L. (2018). De novo assembly of the zucchini genome reveals a whole-genome duplication associated with the origin of the Cucurbita genus. Plant Biotechnol. J..

[26] Sun H., Wu S., Zhang G., Jiao C., Guo S., Ren Y., Zhang J., Zhang H., Gong G., Jia Z. (2017). Karyotype Stability and Unbiased Fractionation in the Paleo-Allotetraploid Cucurbita Genomes. Mol. Plant.

[27] Wu S., Shamimuzzaman M., Sun H., Salse J., Sui X., Wilder A., Wu Z., Levi A., Xu Y., Ling K.S., Fei Z. (2017). The bottle gourd genome provides insights into Cucurbitaceae evolution and facilitates mapping of a Papaya ring-spot virus resistance locus. Plant J. Cell Mol. Biol..

[28] Valmonte G.R., Arthur K., Higgins C.M., MacDiarmid R.M. (2014). Calcium-dependent protein kinases in plants: evolution, expression and function. Plant Cell Physiol..

[29] Zheng Y., Wu S., Bai Y., Sun H., Jiao C., Guo S., Zhao K., Blanca J., Zhang Z., Huang S. (2019). Cucurbit Genomics Database (CuGenDB): A central portal for comparative and functional genomics of cucurbit crops. Nucleic Acids Res..

[30] Liu W., Li W., He Q., Daud M.K., Chen J., Zhu S. (2014). Genome-wide survey and expression analysis of calcium-dependent protein kinase in Gossypium raimondii. PLoS ONE.

[31] Vision T.J., Brown D.G., Tanksley S.D. (2000). The origins of genomic duplications in Arabidopsis. Science.

[32] Cannon S.B., Mitra A., Baumgarten A., Young N.D., May G. (2004). The roles of segmental and tandem gene duplication in the evolution of large gene families in Arabidopsis thaliana. BMC Plant Biol..

[33] Coca M., San Segundo B. (2010). AtCPK1 calcium-dependent protein kinase mediates pathogen resistance in Arabidopsis. Plant J. Cell Mol. Biol..

[34] Zou J.J., Wei F.J., Wang C., Wu J.J., Ratnasekera D., Liu W.X., Wu W.H. (2010). Arabidopsis calcium-dependent protein kinase CPK10 functions in abscisic acid- and Ca2+-mediated stomatal regulation in response to drought stress. Plant Physiol..

[35] Li Z., Takahashi Y., Scavo A., Brandt B., Nguyen D., Rieu P., Schroeder J.I. (2018). Abscisic acid-induced degradation of Arabidopsis guanine nucleotide exchange factor requires calcium-dependent protein kinases. Proc. Natl. Acad. Sci. USA.

[36] Zuo R., Hu R., Chai G., Xu M., Qi G., Kong Y., Zhou G. (2013). Genome-wide identification, classification, and expression analysis of CDPK and its closely related gene families in poplar (Populus trichocarpa). Mol. Biol. Rep..

[37] Kong X., Lv W., Jiang S., Zhang D., Cai G., Pan J., Li D. (2013). Genome-wide identification and expression analysis of calcium-dependent protein kinase in maize. BMC Genom..

[38] Xu G., Guo C., Shan H., Kong H. (2012). Divergence of duplicate genes in exon-intron structure. Proc. Natl. Acad. Sci. USA.

[39] Roelfsema M.R.G., Hedrich R. (2005). In the light of stomatal opening: New insights into ‘the Watergate’. New Phytol..

[40] Xu J., Tian Y.-S., Peng R.-H., Xiong A.-S., Zhu B., Jin X.-F., Gao F., Fu X.-Y., Hou X.-L., Yao Q.-H. (2010). AtCPK6, a functionally redundant and positive regulator involved in salt/drought stress tolerance in Arabidopsis. Planta.

[41] Yang Y., Mo Y., Yang X., Zhang H., Wang Y., Li H., Wei C., Zhang X. (2016). Transcriptome Profiling of Watermelon Root in Response to Short-Term Osmotic Stress. PLoS ONE.

[42] Gutermuth T., Lassig R., Portes M.-T., Maierhofer T., Romeis T., Borst J.-W., Hedrich R., Feijó J.A., Konrad K.R. (2013). Pollen tube growth regulation by free anions depends on the interaction between the anion channel SLAH3 and calcium-dependent protein kinases CPK2 and CPK20. Plant Cell.

[43] Edgar R.C. (2004). MUSCLE: Multiple sequence alignment with high accuracy and high throughput. Nucleic Acids Res..

[44] Wang Y., Tang H., Debarry J.D., Tan X., Li J., Wang X., Lee T.H., Jin H., Marler B., Guo H. (2012). MCScanX: A toolkit for detection and evolutionary analysis of gene synteny and collinearity. Nucleic Acids Res..

[45] Mo Y., Yang R., Liu L., Gu X., Yang X., Wang Y., Zhang X., Li H. (2016). Growth, photosynthesis and adaptive responses of wild and domesticated watermelon genotypes to drought stress and subsequent re-watering. Plant Growth Regul..

[46] Song Q., Li D., Dai Y., Liu S., Huang L., Hong Y., Zhang H., Song F. (2015). Characterization, expression patterns and functional analysis of the MAPK and MAPKK genes in watermelon (Citrullus lanatus). BMC Plant Biol..

[47] Jing-Hua Y., Yuan G., Yan-Man L., Xiao-Hua Q., Zhang M.-F. (2008). Salicylic acid-induced enhancement of cold tolerance through activation of antioxidative capacity in watermelon. Sci. Hortic..

[48] Yang X., Li H., Yang Y., Wang Y., Mo Y., Zhang R., Zhang Y., Ma J., Wei C., Zhang X. (2018). Identification and expression analyses of WRKY genes reveal their involvement in growth and abiotic stress response in watermelon (Citrullus lanatus). PLoS ONE.

[49] Kong Q., Yuan J., Gao L., Zhao S., Jiang W., Huang Y., Bie Z. (2014). Identification of suitable reference genes for gene expression normalization in qRT-PCR analysis in watermelon. PLoS ONE.

[50] Livak K.J., Schmittgen T.D. (2001). Analysis of relative gene expression data using real-time quantitative PCR and the 2^−ΔΔCt^ Method. Methods.

