# Supplementary material for: Comparative Analysis of Calcium-Dependent Protein Kinase in Cucurbitaceae and Expression Studies in Watermelon
Source: Int J Mol Sci. 2019 May 23;20(10):2527. doi: 10.3390/ijms20102527 (PMC6566760; doi:10.3390/ijms20102527)
Supplement: Supplementary file 1 [file ijms-20-02527-s001.zip › ijms-479077 suppl/ijms-479077 Supplementary figures.pdf]

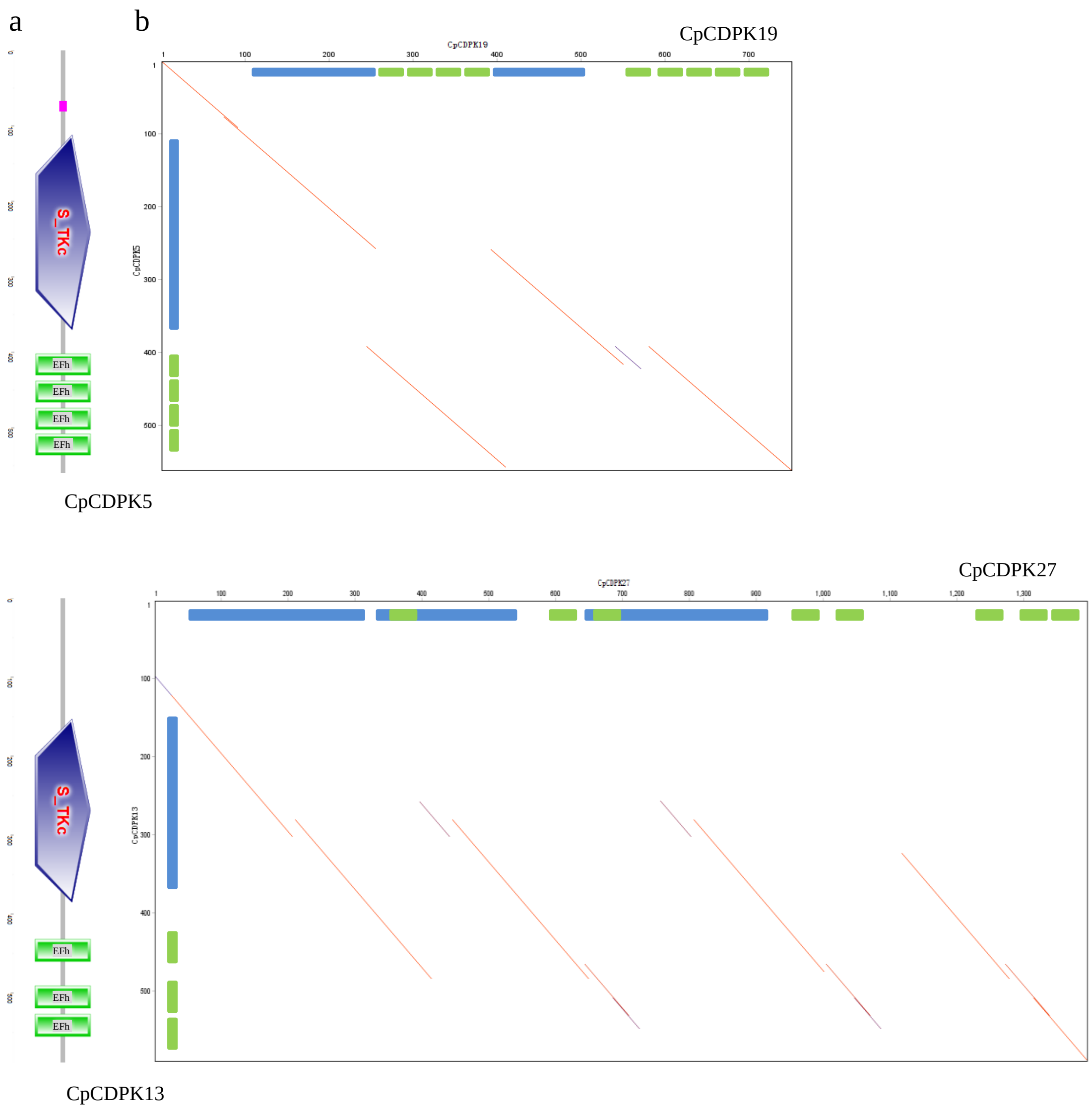

Figure S1. Domain prediction and Dot plot analysis of four CpCDPKs. (**a**) Domain composition of CpCDPK5 and CpCDPK13; (**b**) Dot plot analyses of CpCDPK homologs. Blue and green rectangles represent STKs_CAMK protein kinase and EF-hand domain, respectively.

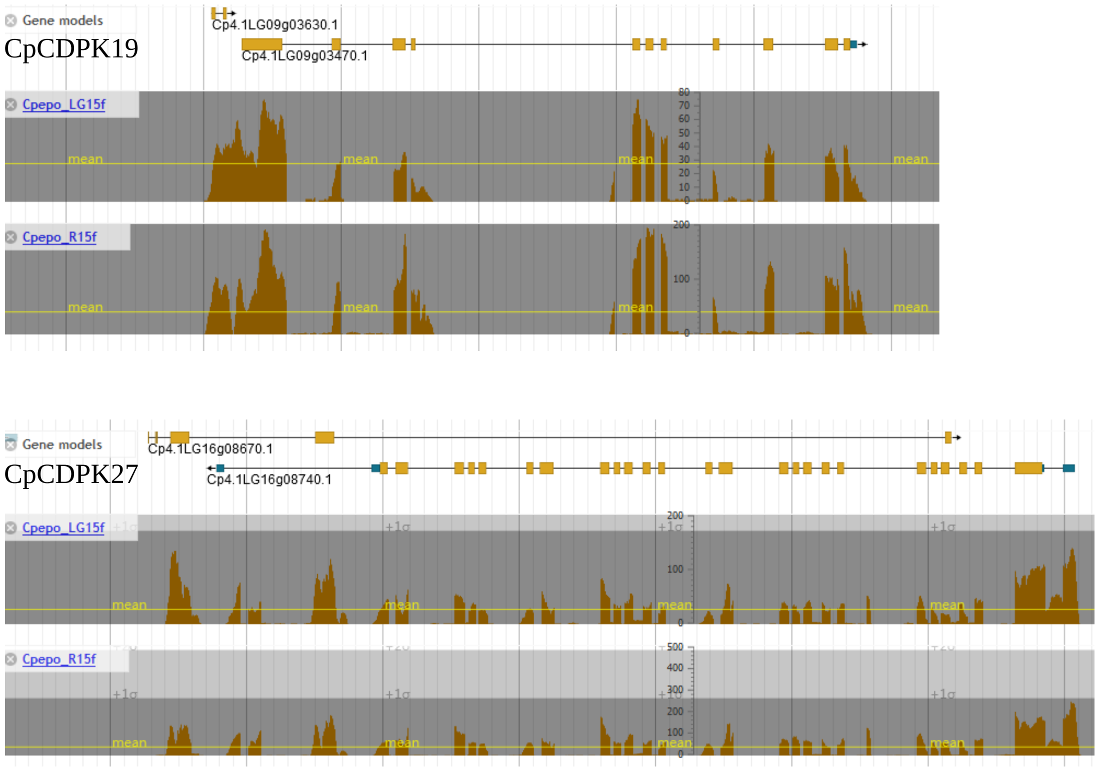

Figure S2. Expression levels of *CpCDPK19* and *CpCDPK27* using transcriptome data (BioProject: PRJNA339848) on the Cucurbit Genomics Database. LG: oilseed pumpkin, 'Lady Godiva'; R: acorn squash, 'Sweet REBA'; f: fruit; 15: 15 days after pollination.

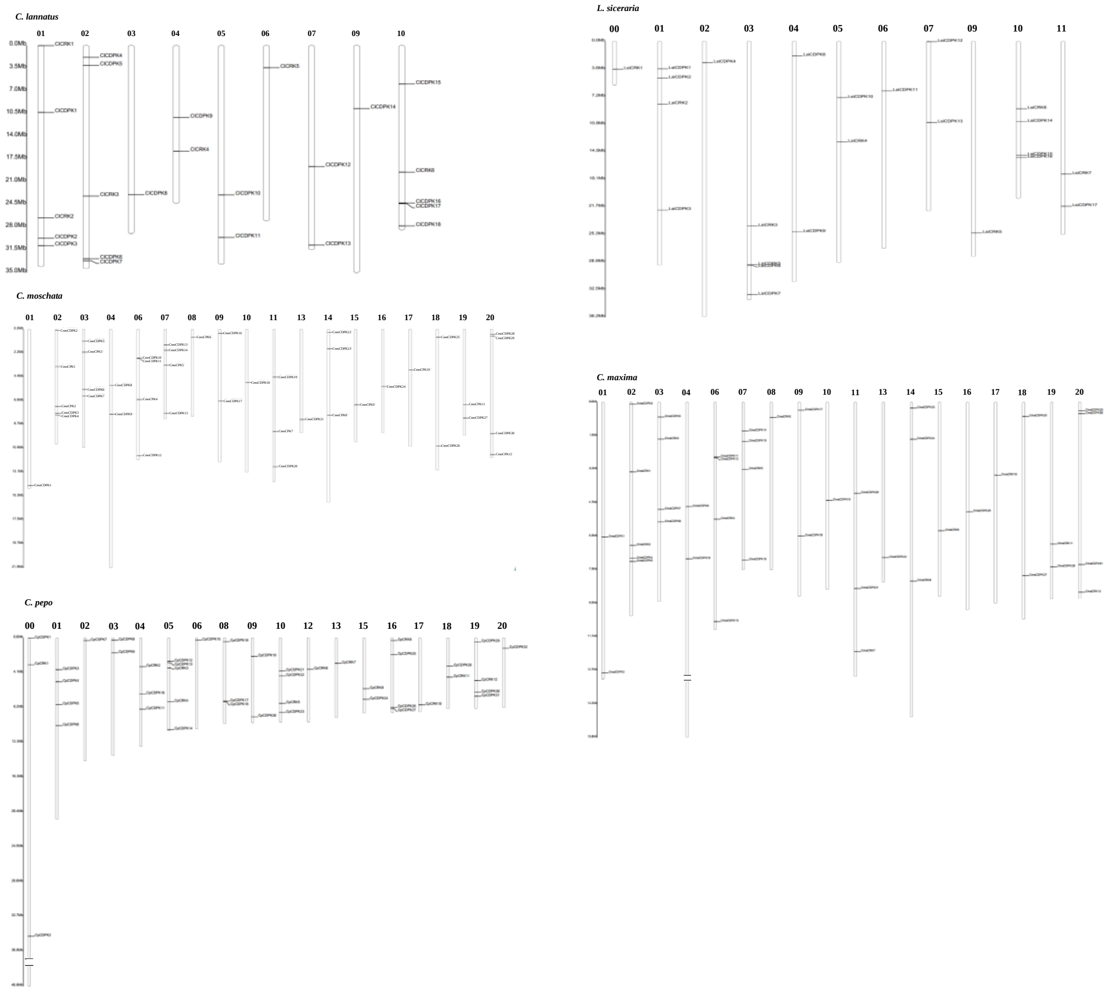

Figure S3. Chromosomal distributions of *CDPK* and *CRK* genes in five Cucurbitaceae species.

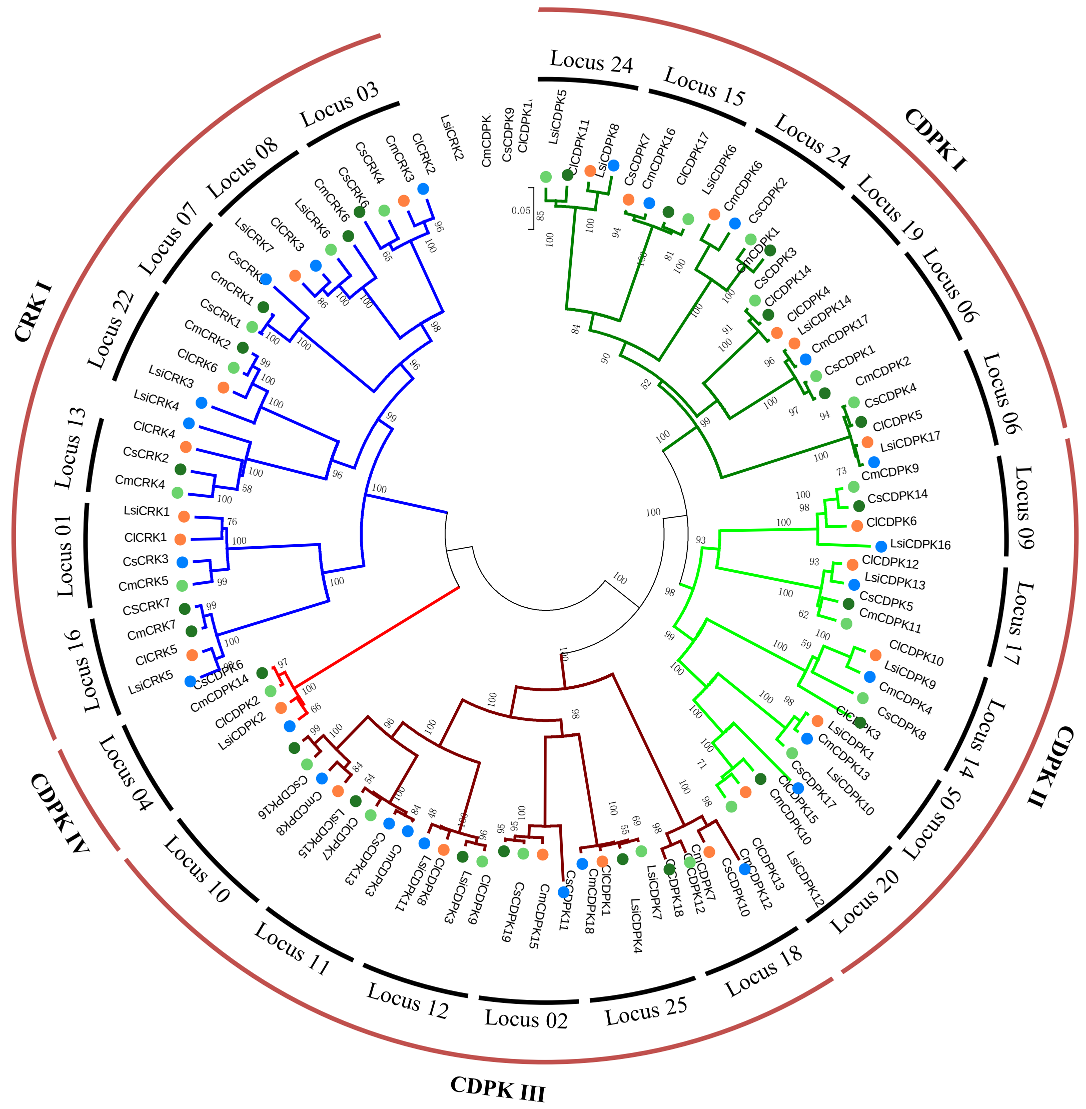

Figure S4. Phylogenetic tree of CDPKs and CRKs from four species in the Benincaseae tribe.

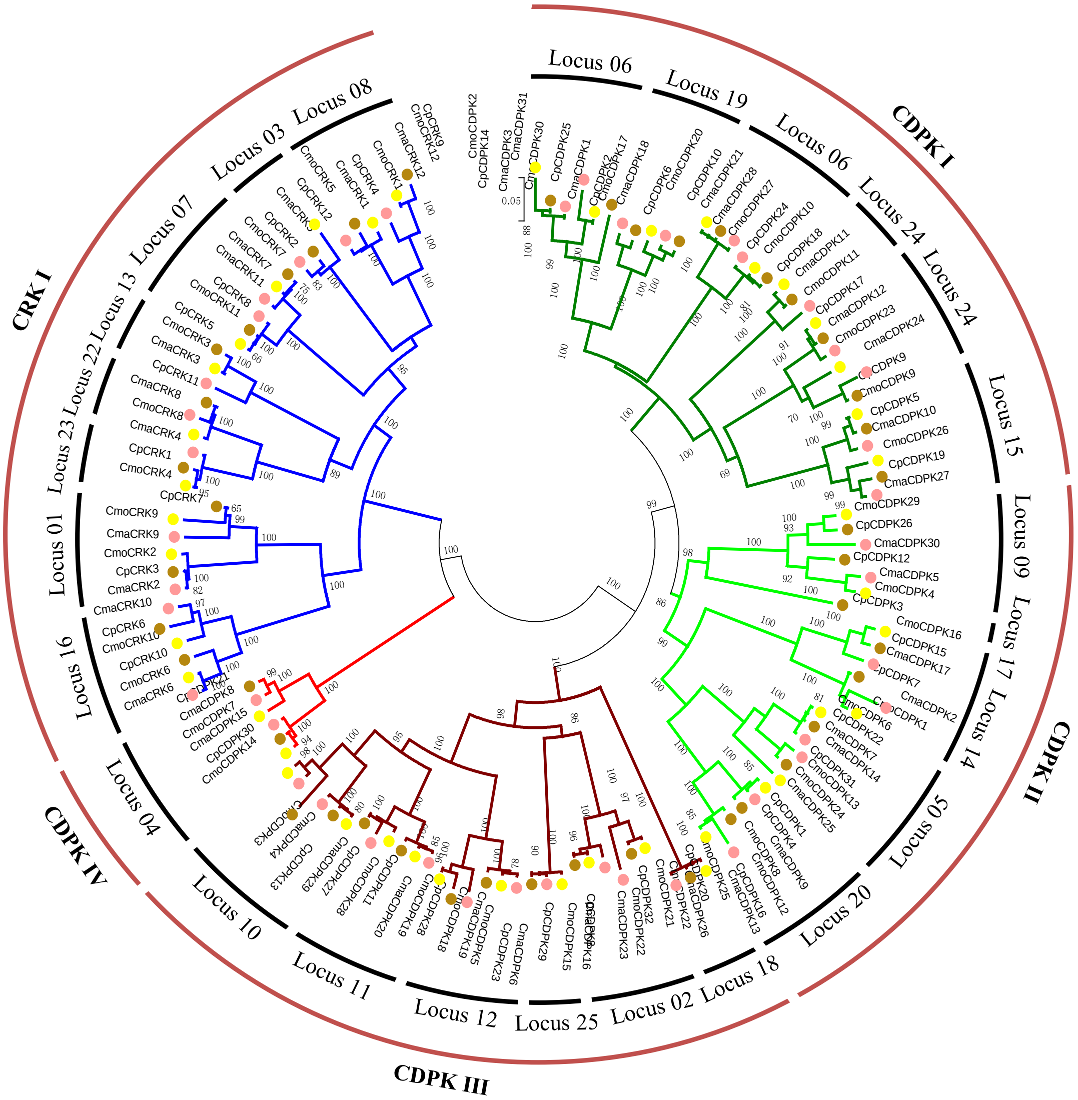

Figure S5. Phylogenetic tree of CDPKs and CRKs from three species in the Cucurbiteae tribe.

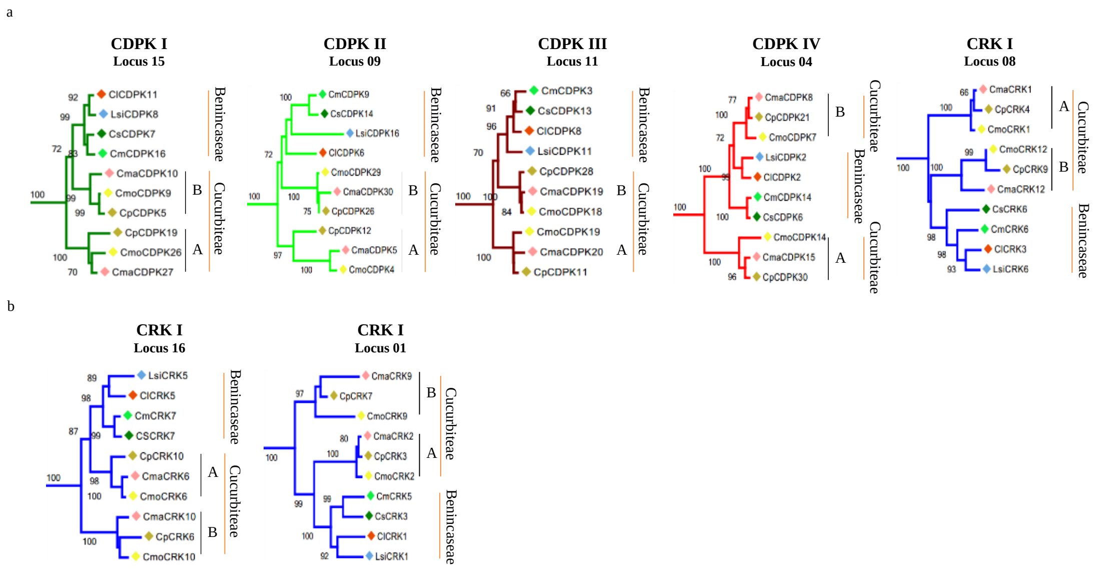

Figure S6. Detailed topologies of seven loci in Figure 3. (**a**) Compared to sub-genome A, CDPK/CRK homologs from sub-genome B were clustered with that from Benincaseae genus; (**b**) CRKs from sub-genome A showed close relationships with that from the Benincaseae genus in loci 01 and 16.

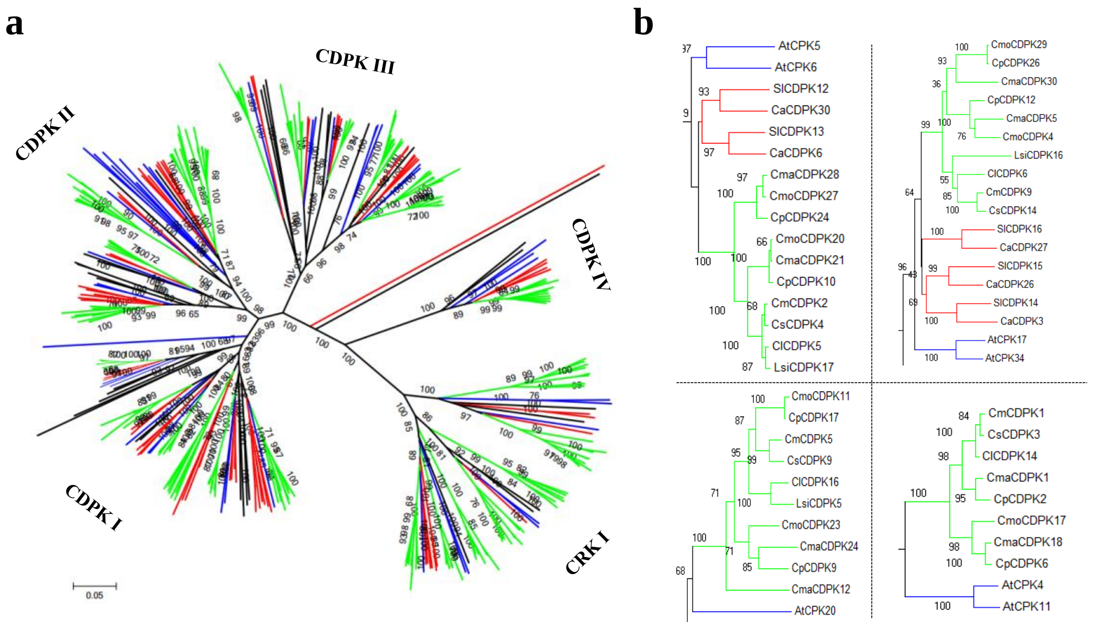

Figure S7. Phylogenetic tree constructed with CDPK and CRK homologs from four families (a). Homologs from Cucurbitaceae (including six species), Solanaceae (including tomato and pepper), Cruciferae (*Arabidopsis*), and Poaceae (rice) were marked in green, red, blue, and black, respectively;(b) Magnified views of four clades in phylogenetic tree (a).

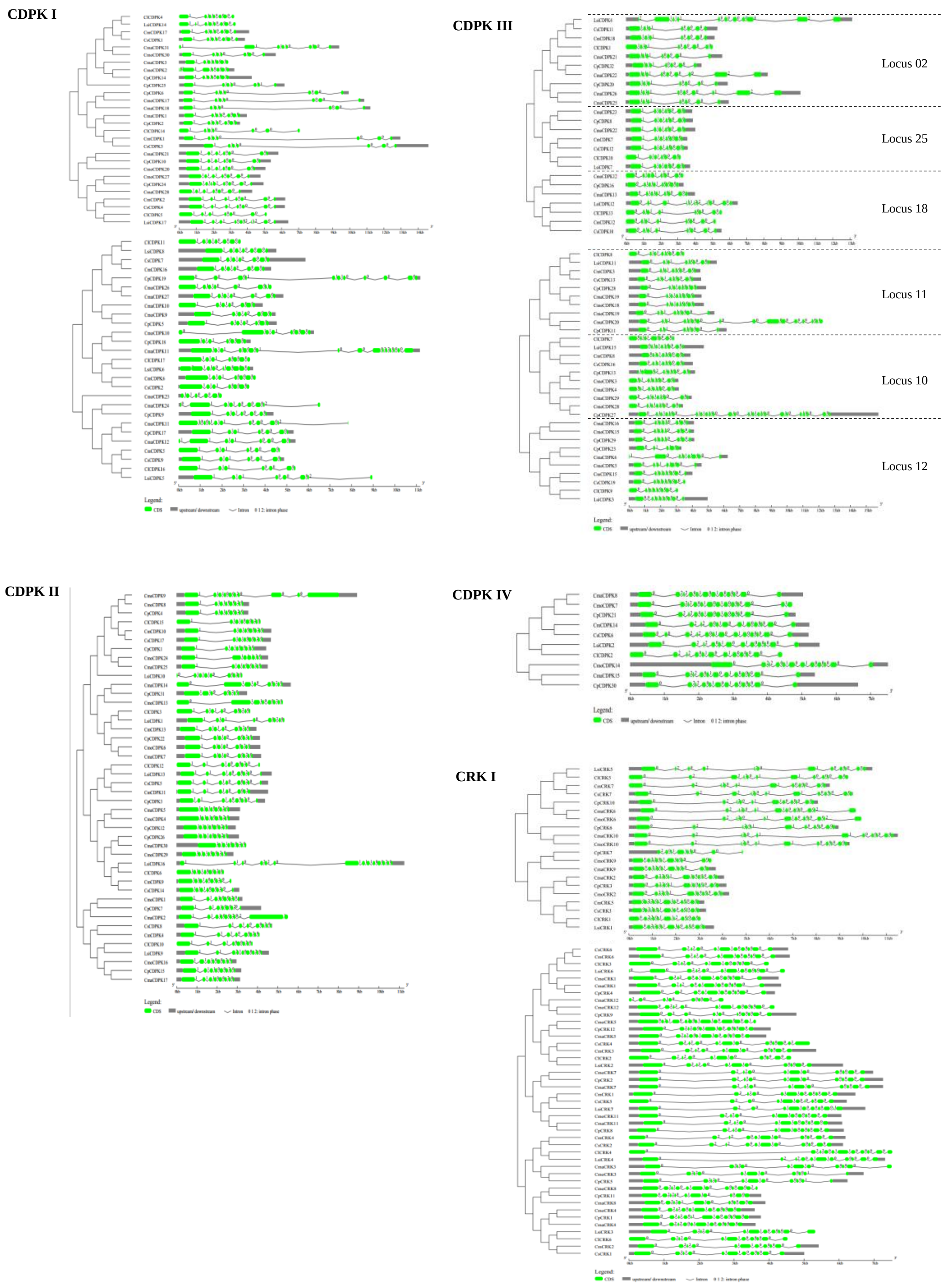

Figure S8. Exon-intron organizations of CDPKs and CRKs in Cucurbitaceae.
